# Antioxidative Defense Genes and Brain Structure in Youth Bipolar Disorder

**DOI:** 10.1093/ijnp/pyab056

**Published:** 2021-08-13

**Authors:** Yi Zou, Kody G Kennedy, Anahit Grigorian, Lisa Fiksenbaum, Natalie Freeman, Clement C Zai, James L Kennedy, Bradley J MacIntosh, Benjamin I Goldstein

**Affiliations:** 1 Department of Pharmacology, University of Toronto, Toronto, ON, Canada; 2 Centre for Youth Bipolar Disorder, Centre for Addiction and Mental Health, Toronto, ON, Canada; 3 Psychiatric Neurogenetics Section, Campbell Family Mental Health Research Institute, Centre for Addiction and Mental Health, Toronto, ON, Canada; 4 Department of Psychiatry, University of Toronto, Toronto, ON, Canada; 5 Heart and Stroke Foundation, Canadian Partnership for Stroke Recovery, Sunnybrook Research Institute, Toronto, ON, Canada; 6 Department of Medical Biophysics, University of Toronto, Toronto, ON, Canada; 7 Hurvitz Brain Sciences Program, Sunnybrook Research Institute, Toronto, ON, Canada

**Keywords:** Bipolar disorder, brain structure, rs4880, rs3792797, youth

## Abstract

**Background:**

Oxidative stress is implicated in the neuropathology of bipolar disorder (BD). We investigated the association of single-nucleotide polymorphisms (SNPs) in the antioxidative genes superoxide dismutase 2 (*SOD2*) and glutathione peroxidase 3 (*GPX3*) with structural neuroimaging phenotypes in youth BD.

**Methods:**

*SOD2* rs4880 and *GPX3* rs3792797 SNP genotypes, along with structural magnetic resonance imaging, were obtained from 147 youth (BD = 75; healthy controls = 72). Images were processed using FreeSurfer, yielding surface area, volume, and thickness values for regions of interest (prefrontal cortex [PFC], caudal anterior cingulate cortex, hippocampus) and for vertex-wise whole-brain analysis. Analyses controlled for age, sex, race, and intracranial volume for volume, area, and thickness analyses.

**Result:**

Regions of interest analyses revealed diagnosis-by-*SOD2* rs4880 interaction effects for caudal anterior cingulate cortex volume and surface area as well as PFC volume; in each case, there was lower volume/area in the BD GG genotype group vs the healthy controls GG genotype group. There was a significant BD diagnosis × *GPX3* rs3793797 interaction effect for PFC surface area, where area was lower in the BD A-allele carrier group vs the other genotype groups. Vertex-wise analyses revealed significant interaction effects in frontal, temporal, and parietal regions related to smaller brain structure in the BD *SOD2* rs4880 GG group and BD *GPX3* rs3793797 A-allele carrier group.

**Conclusion:**

We found preliminary evidence that *SOD2* rs4880 and *GPX3* rs3792797 are differentially associated with brain structures in youth with BD in regions that are relevant to BD. Further studies incorporating additional neuroimaging phenotypes and blood levels of oxidative stress markers are warranted.

**Significance Statement**

The current study, focused on youth early in their course of bipolar disorder, provides an opportunity to examine genetic effects early in the course of illness. Both the regions-of-interest and whole-brain approaches revealed that the association of antioxidative genes with brain structure in youth differs between those with and without bipolar disorder. This finding addresses a gap in the current literature regarding the intermediate phenotypes related to antioxidative defense system, which is known to be aberrant in bipolar disorder. Future longitudinal studies incorporating peripheral biomarkers are needed to shed light on the long-term impact of these antioxidative genes on brain structure and cognition. Ultimately, this line of research advances knowledge that may in the future inform clinical decisions, such as the use of antioxidative interventions.

## Introduction

Bipolar disorder (BD) is a severe psychiatric disorder that affects approximately 2%–5% of the population worldwide (Jann, 2014). BD is polygenic and is thought to result from the interaction of environmental factors with multiple genes of small to moderate effect (Kieseppä, 2004; Purcell, 2009). The disorder has an estimated heritability of approximately 85%, which is among the highest of psychiatric disorders (Uher, 2009). Oxidative stress has been proposed to play an essential role in the neuropathology of BD (Andreazza, 2008; Ng, 2008; Frey, 2013). A recent candidate gene study with 325 adults with BD and 392 healthy control (HC) adults identified that an interaction of 2 antioxidative enzyme single nucleotide polymorphisms (SNPs), superoxide dismutase 2 (*SOD2*) rs4880 and glutathione peroxidase 3 (*GPX3*) rs3792797, was associated with an increased risk for BD (Fullerton, 2010). In a prior study, our group found that *GPX3* rs3792797 was associated with BD diagnosis in a case-control study of youth (Dimick, 2020).

Oxidative stress is defined as an accumulation of free radicals in the body due to an imbalance between oxidant generation and antioxidant capacity (Ghosh, 2018). Reactive oxygen species (ROS) such as superoxide radical (O_2_^•-^), hydrogen peroxide (H_2_O_2_), and hydroxyl radical (•OH) are oxygen-containing free radicals that are produced as either a by-product of the electron transport chain or as the final products of antioxidative enzyme activity (Ghosh, 2018). Antioxidative enzymes such as superoxide dismutase (SOD), catalase, and glutathione peroxidase (GPx) are encoded in the human genomes and play an essential in eliminating ROS (Ghosh, 2018). Genetic variation of antioxidative enzymes can affect their expression and function (Shimoda-Matsubayashi, 1996; Forsberg, 2001). The brain is highly susceptible to oxidative stress; as such, deficits in antioxidative capacity are particularly damaging to the brain (Murphy, 2009; Ghosh, 2018).

Previous peripheral biomarker and post-mortem brain studies have demonstrated both an increase in oxidative stress and a decrease in antioxidant capacity in both the peripheral blood and brain of adult BD patients compared with HC (Brown, 2014). Results from adult BD post-mortem brain studies have shown an increase in oxidative stress in the hippocampus and several frontal brain regions, such as the anterior cingulate cortex (ACC) and the prefrontal cortex (PFC) (Wang, 2009; Andreazza, 2010; Che, 2010; Gawryluk, 2011). Importantly, these cortical and subcortical brain regions are known to be relevant to BD (Blumberg, 2003; Bora, 2010; Ellison-Wright and Bullmore, 2010; Hajek, 2012; Janssen, 2014; Hanford, 2016; Ganzola and Duchesne, 2017; Lu, 2019). However, to the extent of our knowledge, no prior study has examined the association of *SOD2* rs4880 or *GPX3* rs379279 SNPs with neuroimaging phenotypes in any population.

The *SOD2* gene, located on chromosome 6q5, encodes functional SOD2 enzyme (also referred to as manganese superoxide dismutase, MnSOD) that mainly functions in the mitochondria (Bresciani, 2015). The rs4880 SNP, a substitution of A to G, results in the switch of valine (Val) to an alanine (Ala) at position 16 (Bag and Bag, 2008). A large multi-site study with 923 schizophrenia inpatients found that *SOD2* rs4880 G-allele carriers showed poorer attention in the schizophrenia group compared with the AA homozygous group (Zhang, 2014). In contrast, other studies have reported the *SOD2* rs4880 A allele is associated with higher risk of schizophrenia and depression compared with the G allele (Hori, 2000; Cumurcu, 2013; Wigner, 2018). The *GPX3* rs3792797 SNP that encodes the extracellular GPX3 enzyme is located on chromosome 5 with A as the minor allele (Pae, 2006). This SNP has not been previously linked to any other psychiatric disorders, but a previous study has suggested a protective effect of GPX3 during brain maturation and aging (Kim, 2009).

In summary, genetic factors underlying antioxidative capacity, including *SOD2* rs4880 and *GPX3* rs3792797 SNPs, may be relevant to BD-related neurostructural endophenotypes. Thus, the current study aims to examine SOD2 rs4880 GG genotype and GPX3 rs3792797 in relation to structural neuroimaging phenotypes in youth with and without BD. Examining this relationship in the youth population offers the advantage of providing a glimpse into putative genetic effects early in the course of illness, with fewer years of exposure to the symptoms, lifestyle, medical comorbidity, and treatment of BD (McEwen, 1998; Kapczinski, 2008). Based on the available sample size, we examined *SOD2* rs4880 (AA, AG, GG) and *GPX3* rs3792797 (AA/AC, CC) separately. We hypothesized interaction such that the association of these genotypes with structural neuroimaging phenotypes would differ for youth with BD vs HC. Pre-specified regions of interest (ROI) analyses, based on prior studies linking these regions with BD and/or oxidative stress, were complemented by whole brain voxel-wise analyses (Blumberg, 2003; Bearden, 2008; Wang, 2009; Andreazza, 2010; Che, 2010; Gawryluk, 2011; Hajek, 2012; Hanford, 2016; Lu, 2019; Wang, 2019).

## MATERIALS AND METHODS

### Participants

The current study included 147 participants, of which 2 participants were missing *GPX3* and 2 participants were missing *SOD2* genotype data. As a result, 145 participants were analyzed for each SNP. Of these 147 participants, 91 were also previously included in the aforementioned genetics study that did not include neuroimaging (Dimick, 2020). BD participants with a diagnosis of BD-I (bipolar I disorder), BD-II (bipolar II disorder), or BD-NOS (bipolar-not other specified), were recruited from a tertiary subspecialty clinic at an academic health sciences center. HC were recruited via hospital and community advertisements. All participants were English speaking between the ages of 13 and 20 years old. The study protocol was approved by the Sunnybrook Research Ethics Board, and written informed consent was obtained from all participants and 1 of their guardians prior to participation. Participants were excluded for any of the following reasons: contraindications to magnetic resonance imaging (MRI); pre-existing cardiac, autoimmune and/or inflammatory illness; taking anti-inflammatory, anti-platelet, anti-lipidemic, anti-hypertensive, or hyperglycemic agents; substance dependence in the past 3 months. HC were excluded if they had lifetime mood or psychotic disorders, anxiety disorders, or a family history of BD or psychotic illnesses.

### Psychiatric and Anthropometric Measures

Diagnosis and Statistical Manual of Mental Disorders, fourth edition (DSM-IV) version of Schedule for Affective Disorders and Schizophrenia for School Age Children, Present and Lifetime version (KSADS-PL) was employed in the current study as KSADS-PL for DSM5 was published in 2016, well after recruitment had begun (participants were enrolled from 2012–2019) (Kaufman, 1997). KSADS-PL is a semi-structured diagnostic interview and was used to evaluate psychiatric diagnoses (including BD and comorbid substance use disorders) for all participants (Kaufman, 1997). The KSADS Depression Rating Scale and the Mania Rating Scale were used to assess related diagnosis and mood symptom severity scores (Chambers, 1985; Axelson, 2003). BD-I and BD-II were defined using DSM-IV. BD-NOS was defined using criteria previously operationalized by the Course and Outcome of Bipolar Illness in Youth study: these include: (1) 2 DSM-IV–confirmed manic symptoms; (2) a minimum of 4 cumulative 24-hour periods of episodes with elevated/irritable mood for at least 4 hours during each episode; and (3) change in functioning (Axelson, 2006). Information regarding ancestry was collected via self-report. Psychotropic medication use and tobacco use were collected during the K-SADS-PL interview. All interviewers in the current study were trained on the KSADS-PL under the supervision of a licensed child-youth psychiatrist (B.I.G.).

Height (cm) and weight (kg) were collected twice from each participant and averaged for accuracy consideration via following the standard procedure (Krebs, 2007). Body mass index (BMI) was computed as weight/height^2^.

### Saliva and DNA Extraction

All participants were instructed to refrain from eating, drinking, smoking, and chewing gum 30 minutes prior to saliva collection. DNA Genotek Oragene-500 (DNA Genotek Inc, Ottawa, Canada) kits were used to collect saliva samples (2 mL) from each participant. DNA extraction was performed in the Neurogenetics Laboratory at the Centre for Addiction and Mental Health (Toronto, Canada) using a chemagic MSM I DNA extractor (Perkin-Elmer, Waltham, MA, USA) per manufacturer instructions. DNA was quantified using Nanodrop 8000 spectrophotometer (ThermoFisher Scientific, Waltham, MA, USA) and diluted to achieve a final concentration of 20 ng/µL.

### Genotyping

The *SOD2* rs4880 and *GPX3* rs3792797 SNPs were genotyped using the TaqMan Format32 method (ThermoFisher Scientific) per manufacturer instructions. A custom assay for the Amelogenin region was included for the purpose of quality control. Briefly, 2 µL of extracted DNA sample was combined with 2 µL of 2X TaqMan Open Array Master Mix. The mixture was loaded onto the Open Array genotyping plates using the AccuFill System (ThermoFisher Scientific). The genotyped data were imported into the TaqMan Genotyper software version 1.3 and manually confirmed by 2 independent researchers. Hardy-Weinberg Equilibrium analysis was performed to examine potential sampling bias using the PLINK software version 1.90, and no violation of the Hardy-Weinberg equilibrium (i.e., all *P* > .05) was reported (Hosking, 2004; Purcell S, 2007). Technicians were blinded to diagnosis.

### MRI Acquisition

Structural images of the brain were collected on a research-dedicated 3 Tesla Philips Achieva MRI scanner (Philips Medical Systems, Best, Netherlands). The acquisition used the body coil for signal transmission and 8-channel head coil for receiving the signal. The T1-weighted high resolution fast-field echo imaging with the following parameters was used: repetition time of 9.5 milliseconds (ms), echo time of 2.3 ms, inversion time of 1400 ms, spatial resolution of 0.94 × 1.17 × 1.2 mm, acquisition matrix of 256 × 164 × 140, field of view of 240 × 191 mm, flip angle of 8°, and scan duration of 8 minutes and 56 seconds.

### Imaging Processing

Three-dimensional reconstruction of the T_1_-weighted images was performed using FreeSurfer (V6.0) software. The 3-dimensional images were quality-controlled for head motion or other artifacts prior to further processing. Briefly, the processing steps include automated skull stripping, field inhomogeneity correction, and registration to the Montreal Neurological Institute (MNI305) atlas (Fischl Bruce, 2012). Automated segmentation classified subcortical structures and cortical white and grey matter. This process includes triangular tessellation (triangle-based mesh) generation, smoothing, and topology correction (Fischl B, 2001; Segonne, 2007). Finally, cortical parcellation was completed via registering each participant’s inflated brain to a canonical template, which allowed anatomical alignment of the brain. The registered brain was then mapped to the FreeSurfer default Desikan-Killiany probabilistic atlas to label 34 gyral regions of interest per hemisphere (Fischl Bruce, 2004).

### Statistical Analyses

Statistical analyses were performed using the SPSS statistic software (IBM Corp; New York, NY, USA), version 26 for clinical and demographic variables. Shapiro-Wilks test and Levene’s test were used to check normality and equal variance assumptions of all continuous variables. Group differences were evaluated using a 2-way ANOVA for continuous variables and chi-square (χ ^2^) tests for categorical variables.

Neuroimaging-genetic analyses were completed using MATLAB version R2018b. Main effects for genotype and diagnosis and diagnosis-by-genotype interaction effects, for PFC, caudal ACC (cACC), and hippocampus ROI analyses were tested using a General Linear Model with age, sex, race, and intracranial volume (ICV) as covariates. ICV was not included as a covariate in the model for cortical thickness analysis as ICV does not explain cortical thickness (Liem, 2015). Family-wise Bonferroni correction was used to correct for multiple ROI comparisons (α = .05/3 = .017). For the whole-brain vertex-wise exploratory analyses, a 10-mm kernel of full width at half-maximum was used in brain surface smoothing before mapping volumetric, surface area, and thickness data to the canonical template. Vertex-wise contrasts for genotype main effect and diagnosis-by genotype interaction effect for each brain structure measurement was entered into the General Linear Model together with the aforementioned covariates. The significance level was set at *P* < .05, and results were corrected for multiple comparisons using Monte-Carlo simulation threshold at a log10 *P* value of .05. Cluster-wide *P* values were then calculated as the probability of seeing a cluster of that size and reported for each significant cluster. Post-hoc analyses were conducted for ROI and vertex-wise whole brain analyses in regions that revealed significant SNP main effect and/or diagnosis-by-SNP interaction effect.

## RESULTS

### Demographics and Clinical Characteristics

Demographic characteristics are summarized in Tables 1 and 2. There were no significant age or sex differences between BD and HC participants. For the *SOD2* rs4880 genotype comparison, participants in the BD group were more likely to be of European ancestry and had higher BMI compared with HC participants. For the *GPX3* rs3792797 genotype comparison (which differed from SOD2 slightly in terms of subgroup size), BMI was higher for the BD group compared with HC. In addition, there were significant differences for race across the *SOD2* genotype groups, explained by a higher proportion of participants of European ancestry in the AG genotype group compared with the AA and GG groups. Clinical characteristics for the BD group are summarized in Table 2. In the current sample, 17 (23%) BD participants had lifetime substance use disorder, of these 17% has current drug/alcohol abuse. No BD participant has current drug/alcohol dependence.

**Table 1.** Demographic Characteristics by Diagnosis and Oxidative Defense Genotypes

		BD AA (n = 20)	BD AG (n = 40)	BD GG (n = 14)	HC AA (n = 22)	HC AG (n = 35)	HC GG (n = 14)
*SOD2* rs4880	Age	17.17 ± 0.36	17.51 ± 0.25	17.02 ± 0.43	17.20 ± 0.34	16.99 ± 0.27	17.00 ± 0.43
	Sex (n, % female)	11 (55%)	25 (63%)	9 (64%)	11 (50%)	18 (51%)	7 (50%)
	Race (n, % Caucasian)^*a*^^,^^*b*^	13 (65%)	32 (80%)	11 (79%)	8 (36%)	25 (71%)	9 (64%)
	BMI (adjusted)^*a*^	23.17 ± 0.85	24.73 ± 0.60	22.68 ± 1.02	21.17 ± 0.81	21.80 ± 0.65	22.61 ± 1.02
	Tanner Stage	4.20 ± 0.70	4.45 ± 0.64	4.57 ± 0.65	4.14 ± 0.56	4.20 ± 0.68	4.31 ± 0.75
		BD CC (n = 53)	BD AA/AC* (n = 22)		HC CC (n = 42)	HC AA/AC (n = 28)	
*GPX3* rs3792797	Age	17.28 ± 0.21	17.49 ± 0.33		16.65 ± 0.24	17.40 ± 0.29	
	Sex (n, % female)	36 (68%)	10 (45%)		20 (48%)	15 (54%)	
	Race (n, % Caucasian)	41 (77%)	16 (72%)		25 (60%)	18 (64%)	
	BMI (adjusted)^*a*^	24.08 ± 0.53	23.36 ± 0.82		21.77 ± 0.59	21.76 ± 0.72	
	Tanner Stage	4.27 ± 0.55	4.47 ± 0.70		4.26 ± 0.71	4.14 ± 0.61	

Abbreviations: BD, bipolar disorder; BMI, body mass index; HC, healthy control.

*AA/AC combined due to limited subgroup size.

Results are reported in mean ± SD unless otherwise specified.

^*a*^Indicates significant differences between diagnostic groups.

^*b*^Indicates significant difference between genotype groups.

**Table 2.** Clinical Characteristics

	BD (n = 75)
BD-I	26 (35%)
BD-II	23 (31%)
BD-NOS	26 (35%)
Age of onset	14.81 ± 2.66
Lifetime clinical characteristics	
Lifetime psychosis	8 (11%)
Lifetime suicide attempts	12 (16%)
Lifetime self-injurious behaviour	38 (51%)
Lifetime suicidal ideation	47 (63%)
Legal history (police contact/arrest)	19 (25%)
Lifetime physical abuse	2 (3%)
Lifetime sexual abuse	3 (4%)
Lifetime any abuse (physical and/or sexual)	4 (5%)
Lifetime psychiatric hospitalization	37 (49%)
Lifetime comorbid diagnoses	
ADHD	37 (49%)
Anxiety disorder	61 (81%)
Number of anxiety disorders	1.63 ± 1.21
Conduct disorder	3 (4%)
Oppositional defiant disorder	19 (25%)
Substance use disorder	17 (23%)
Nicotine use	11 (15%)
Lifetime medications	
Second generation antipsychotics	55 (73%)
Lithium	19 (25%)
Non-SSRI antidepressants	14 (19%)
SSRI antidepressant	25 (33%)
Stimulants	16 (21%)
Current medications	
Second generation antipsychotics	44 (59%)
Lithium	14 (19%)
Non-SSRI antidepressants	4 (5%)
SSRI antidepressants	7 (9%)
Stimulants	5 (7%)
Family psychiatric history	
Mania/hypomania	36 (48%)
Depression	55 (73%)
Anxiety	46 (61%)
ADHD	25 (33%)

Abbreviations: ADHD, attention deficit-hyperactivity disorder; BD, bipolar disorder; HC, healthy control; NOS, not otherwise specified; SSRI, selective serotonin reuptake inhibitor.

Results are reported in mean ± SD or percentage (%) yes unless otherwise specified.

### SOD2 rs4880 Region of Interest Analyses

**Figure 1.**
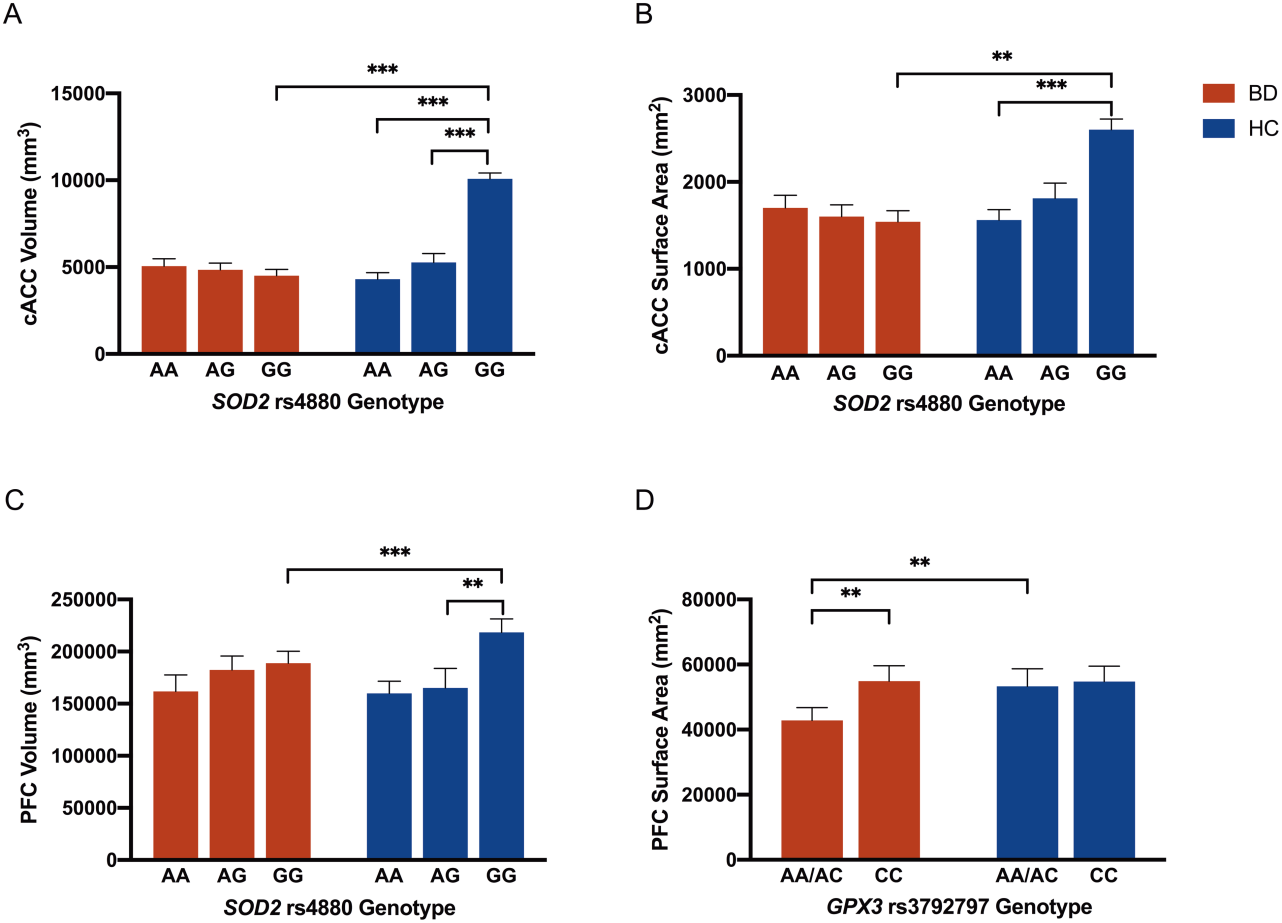
Post-hoc analyses for regions of interest with significant diagnosis-by-genotype interaction effects. *P < .05, **P < .01, ***P < .001. (A–C) Diagnosis-by-superoxide dismutase 2 (*SOD2*) rs4880 interaction effect post-hoc results for caudal anterior cingulate cortex (cACC) volume, cACC surface area, and PFC volume respectively. (D) Diagnosis-by-glutathione peroxidase 3 (*GPX3*) rs3792797 interaction effect post-hoc result for prefrontal cortex (PFC) surface area. Note that only the SOD2 *rs4880* cACC volume finding remained significant after correction for multiple comparisons.

PFC area (F = 4.84; *P* = .03; η ^2^ = 0.04) and volume (F = 8.12; *P* = .01; η ^2^ = 0.06) were significantly smaller in the BD group compared with the HC. PFC area finding did not remain significant after Bonferroni correction. There were significant gene main effects for cACC (F = 5.28, *P* = .01; η ^2^ = 0.04) and PFC (F = 4.91, *P* = .01; η ^2^ = 0.04) volume (Table 3). In each case, the AA and AG groups had smaller brain volume than the GG group (Supplementary 1 and 3). There were also significant interaction effects observed for cACC surface area (F = 3.37, *P* = .04; η ^2^ = 0.03) and volume (F = 7.50, *P < *.01; η ^2^ = 0.06) as well as PFC volume (F = 3.27, *P* = .04; η ^2^ = 0.03) (Table 3; Figure 1A–C). Post-hoc analyses for all reported interaction effects revealed significantly smaller brain structures in the BD GG group compared with the HC GG group. cACC volume and surface area were significantly smaller in the HC AA group compared with the HC GG group (Supplementary 1 and 3). In addition, cACC and the PFC volume were significantly smaller in the HC AG group compared with the HC GG group. For cACC surface area and PFC volume, the interaction effect did not remain significant after Bonferroni correction for multiple comparisons.

**Table 3.** Results for Region of Interests Analyses

		cACC area		cACC volume		cACC thickness		PFC area		PFC volume		PFC thickness		Hippocampal volume	
SNPs		F	P	F	P	F	P	F	P	F	P	F	P	F	P
*SOD2* rs4880	Diagnosis main effect	2.51	.12	0.13	.72	0.03	.86	4.84	**.03**	8.12	**.01** ^*^	1.05	.31	1.37	.24
	Gene main effect	1.89	.16	5.28	**.01** ^*^	0.78	.46	0.78	.46	4.91	**.01** ^*^	1.33	.27	1.98	.14
	Interaction effect	3.37	**.04**	7.50	**.001** ^*^	0.40	.67	1.80	.17	3.27	**.04**	0.42	.66	1.65	.20
*GPX3* rs3792797	Diagnosis main effect	4.22	**.04**	0.44	.51	0.03	.86	1.62	.20	1.71	.19	0.59	.44	0.77	.38
	Gene main effect	2.29	.13	2.67	.11	0.07	.79	7.29	**.008** ^*^	6.11	**.015** ^*^	1.10	.30	4.01	.048
	Interaction effect	0.12	.71	0.46	.50	0.04	.83	4.44	**.04**	2.18	.14	0.11	.75	1.10	.30

Abbreviations: cACC, caudal anterior cingulate cortex; PFC, prefrontal cortex.

*Finding remains significant after correction for multiple comparisons.

Significant gene main effect and interaction effect are bolded.

### GPX3 rs3792797 ROIs Analyses

cACC surface area (F = 4.22; *P* = .04; η ^2^ = 0.03) was significantly smaller in the BD group compared with HC, but the finding did not remain significant after Bonferroni correction. There were significant genotype main effects for PFC, with lower surface area (F = 7.29, *P < *.01; η ^2^ = 0.05) and volume (F = 6.11, *P* = .02; η ^2^ = 0.04) among A-allele carriers compared with CC homozygotes (Table 3). There was also a significant interaction effect for PFC surface area (F = 4.44, *P* = .04; η ^2^ = 0.04), whereby the BD A-allele carrier group had lower surface area compared with the HC A-allele carrier group and the BD CC genotype group (Table 3; Figure 1D; Supplementary 2 and 3). The interaction effect did not remain significant after Bonferroni correction.

### SOD2 rs4880 Vertex-Wise Whole-Brain Analysis

Results of vertex-wise analyses are summarized in Table 4. In terms of main effect, there were 4 significant clusters. For 3 clusters, located in superior temporal, superior frontal, and caudal middle frontal gyri, there was a lower volume in the AA and AG groups compared with the GG group (Supplementary 4). In addition, for superior frontal gyrus, there was lower volume in the AA group compared with the AG group. In the fourth cluster, in the superior temporal gyrus, the AA and AG groups had reduced thickness compared with the GG group (Supplementary 4).

**Table 4.** SOD2 and GPX3 Whole-Brain Analyses

	Cortical measure	Contrast	Peak cluster region	Size of cluster (mm^2^)	cwp	MNI X	MNI Y	MNI Z
*SOD2* rs4880	Area	Interaction effect	rh paracentral gyrus	1815.84	0.008	11.9	−12.5	48.3
	Volume	Gene main effect	lh superior frontal gyrus	1114.53	0.004	−10.0	13.3	52.2
			rh caudal middle frontal gyrus	997.39	0.008	36.8	8.5	39.1
			rh superior temporal gyrus	774.06	0.040	56.3	3.3	−11.7
	Thickness	Gene main effect	rh superior temporal gyrus	1544.18	0.0001	52.1	−7.2	−13.1
*GPX3* rs3792797	Area	Gene main effect	lh caudal middle frontal gyrus	1869.75	0.042	−36.5	13.5	33.7
			lh transverse temporal gyrus	1840.31	0.046	−51.6	−20.4	5.1
			rh rostral middle frontal gyrus	3633.70	0.0004	34.3	28.2	43.0
			rh postcentral gyrus	2417.59	0.009	47.3	−18.6	57.0
		Interaction effect	lh superior frontal gyrus	2839.21	0.002	−8.7	−7.2	57.7
			rh superior frontal gyrus	1991.16	0.028	20.2	24.4	45.6
	Volume	Gene main effect	lh postcentral gyrus	1598.50	0.006	−44.2	−23.3	56.0
			lh caudal middle frontal gyrus	1202.00	0.035	−36.6	17.8	49.8
			rh postcentral gyrus	1661.38	0.001	49.2	−21.1	56.6
		Interaction effect	rh supramarginal gyrus	1056.16	0.043	59.8	−37.8	21.8

Abbreviations: cwp, cluster wide *P* value; lh, left hemisphere; MNI, Montreal Neurological Institute; rh, right hemisphere.

In terms of interaction effect, there was 1 significant cluster, for paracentral gyrus surface area (Table 4); post-hoc analyses revealed a significantly lower surface area in the BD GG group compared with BD AA, BD AG, and HC GG groups (Supplementary 5). In addition, surface area was significantly lower in the HC AA group and the HC AG group compared with the HC GG group (Supplementary 5).

### GPX3 rs3792797 Vertex-Wise Whole-Brain Analysis

Results of vertex-wise analyses are summarized in Table 4. In terms of main effects, there were 4 significant clusters. For 2 clusters, located in caudal middle frontal and postcentral gyri, there was lower cortical volume and surface area in the A-allele carrier group compared with the CC group. For the third and fourth clusters, in the transverse temporal and rostral middle frontal gyrus, the A-allele carrier group showed lower surface area compared with the CC genotype group.

In terms of interaction effects, there were 2 significant clusters in superior frontal and supramarginal gyri for cortical surface area and volume, respectively (Table 4). For the superior frontal gyrus, the significance was due to a lower surface area for BD A-allele carrier group compared with BD CC group and HC A-allele carrier group (Supplementary 6). For the supramarginal gyrus, post-hoc analyses indicated significantly lower cortical volume in the BD A-allele carrier group and HC CC group compared with BD CC group (Supplementary 6). In addition, there was lower volume in the HC CC group compared with the HC A-allele carrier group.

## Discussion

This preliminary study addresses gaps in the literature regarding antioxidative enzyme genes in relation to brain structure in BD and among youth. In ROI analyses, there was a significant interaction effect for *SOD2* rs4880 on cACC volume. For the exploratory whole-brain analysis, significant interaction effects were observed in the temporal lobe for *SOD2* rs4880, and frontal lobe and parietal lobe for *GPX3* rs3792797. Post-hoc analyses revealed smaller regional brain structure in the BD *SOD2* rs4880 GG group (compared with HC GG groups) and the BD *GPX3* rs3792797 A-allele carrier group (compared with the BD CC and HC A-allele carrier groups). Overall, the results indicated that these antioxidative enzyme SNPs were associated with structural brain differences in regions relevant to BD. Although this is not a mechanistic study, the current findings provide inferences regarding the potential implications of redox balance shifting, a result of altered antioxidative enzyme activity/production, to brain structure in BD.

The exact physiological mechanisms explaining how *SOD2* rs4880 and *GPX3* rs3792797 genetic variances influence brain morphology have not yet been fully explored. The smaller brain structure among BD youth with *SOD2* rs4880 GG and *GPX3* rs3792797 A-allele carrier genotypes may relate in part to abnormal neural pruning in early neurodevelopmental stages (Huttenlocher, 1979; Petanjek, 2011). The reorganization process of the brain during pruning is partially regulated by ROS, underscoring the importance of antioxidative defenses as regulators of this process (Cobley, 2018; Sidlauskaite, 2018). The *SOD2* rs4880 SNP is associated with substitution of Val at position 16 to Ala, leading to a conformational change in the secondary structure of SOD2, as the Val allele variant encodes a beta-sheet structure and the Ala allele variant encodes an alpha-helix structure (Shimoda-Matsubayashi, 1996). The switch from a beta-sheet to alpha-helix increases the mobility of this enzyme to move across the mitochondria inner membrane into the matrix, while majority of the Val allele variant encoded SOD2 were imbedded in the inner membrane (Holley, 2012; Bresciani, 2015). Furthermore, the Ala allele encoded SOD2 also has a much higher level of enzymatic activity compared with the Val allele encoded SOD2 (McAtee and Yager, 2010; Bresciani, 2015). Elevated SOD2 level and its enzymatic activity of Ala variant encoded SOD2 in the mitochondria generates increased ROS as a result of SOD2-mediated H_2_O_2_ generation (Shimoda-Matsubayashi, 1997; Bag and Bag, 2008; McAtee and Yager, 2010). When conversion of H_2_O_2_ to water is inefficient, this results in further generation of ROS, specifically hydroxyl radical species (Ghosh, 2018). Such alternation in the brain ROS level might thus impact the neural pruning processes.

In the current study, the BD *SOD2* GG, HC AA, and HC AG groups had significantly lower cACC volume compared with the HC GG group. There is prior evidence of reduced cACC volume in adults with BD (Phillips and Swartz, 2014). The cACC is involved in cognitive control and emotion regulation, which are known to be impaired in BD (Joseph, 2008; Tsitsipa and Fountoulakis, 2015; Heilbronner and Hayden, 2016). The *SOD2* rs4880 G allele and higher SOD2 enzyme activity have been linked to impaired attention in adults with schizophrenia (Zhang, 2014). The reduction in cACC volume of the BD GG group may reflect accelerated cortical pruning due to excessive ROS production (Cobley, 2018). Interestingly, the GG genotype effect was divergent for BD (lower volume) compared with HC (greater volume). This finding might support the Differential Susceptibility Model, which posits that the same genotype that acts advantageously under beneficial environmental conditions can act deleteriously under adverse environmental conditions (Jolicoeur-Martineau, 2019). It is well established that individuals with BD have higher rates of early adversity and life stress, and the symptoms and related behaviors of BD comprise additional stressors (Kapczinski, 2008). Thus, this allostatic load of BD might interact with *SOD2* rs4880 genotype and present phenotypically as neurostructural changes.

Information regarding the impact of *GPX3* rs3792797 SNP on the activity and/or production of its encoded GPX3 enzyme is limited. However, a previous study conducted in healthy individuals across a broad age range reported a positive association of GPX3 gene expression with age in the PFC (*r* = 0.42, *P* = .001), indicating the importance of this antioxidative enzyme in brain oxidative stress protection during brain maturation and aging (Kim, 2009). Moreover, a recent study found elevated GPX3 enzyme levels in cerebrospinal fluid among participants with psychiatric disorders (n = 98, including 27 with BD) compared with controls (Maccarrone, 2013). Taken together, these findings highlight the importance of examining neurostructural correlates of GPX3 in a youth population in the midst of major neurodevelopmental changes.

For the vertex-wise whole-brain analyses, the significant interaction effect for *SOD2* in the right paracentral gyrus was related to lower surface area in the BD GG group compared with HC GG, BD AG, and BD AA groups. Similar to what has been observed in the ROI analyses, the GG genotype effect was divergent for BD (associated with lower surface area) vs HC (associated with higher surface area). The paracentral gyrus, located in the parietal lobe of the brain, is divided into anterior (part of the primary motor cortex) and posterior (part of the primary somatosensory cortex) regions (Spasojević, 2013). A structural neuroimaging meta-analysis of adults with BD-I reported a higher left paracentral gyrus volume in BD vs HC, while no significant finding was observed for the right paracentral gyrus (McCarthy, 2014). However, no prior studies have examined SOD2 effects or interaction effects with diagnosis in adults.

Whole-brain analyses for *GPX3* rs3792797 revealed a significant interaction effect in bilateral superior frontal gyri (SFG) surface area, which was lower in the BD A-allele carrier group than in the BD CC group and HC A-allele carrier group. The SFG is involved in higher cognitive functions such as working memory, which is known to be impaired in BD (Joseph, 2008; Depp, 2012; Li, 2013). Lower SFG volume has been previously reported in individuals with major depressive disorder and in unaffected youth with a family history of BD compared with HC (Cattarinussi, 2019; Kandilarova, 2019). A significant interaction effect was also found for right supramarginal gyrus volume, which was lower in the BD A-allele carrier group and HC CC group compared with the BD CC group. The right supramarginal gyrus plays an essential role in regulating empathy judgment, which is known to be impaired in adults with BD (Shamay-Tsoory, 2009; Silani, 2013; Bodnar and Rybakowski, 2017).

There are several limitations to this study. First, the cross-sectional design precludes inferences regarding the timing of the observed neurostructural differences in relation to the timing of onset of BD. As such, it is unclear whether the observed differences preceded onset of BD or emerged concurrently and/or subsequently to symptom onset. Second, the sample size of the current study, while comparatively large for a neuroimaging study in this field, only allowed for the detection of a medium to large effects while the gene effects are usually small to medium (Kerner, 2014). Moreover, the small sample size limited our ability to conduct subgroup analyses (e.g., BD subtypes, mood states, sex differences, medications) and additive and/or interactive genetic effect analyses. Third, the current study did not include measurement of oxidative stress proteins or neurocognition, which could provide insights about putative mechanisms underlying our observed findings and about whether observed findings are salutary or deleterious.

In summary, the current study showed that the association of *SOD2* rs4880 and *GPX3* rs3792797 SNPs with brain structure in youth differs between those with vs without BD. This finding adds to the current literature regarding the potential role of anomalous antioxidant defense mechanisms in the early stage of disease development, highlighting specific regions that may be particularly susceptible to oxidative stress. Future longitudinal studies are warranted to explore the long-term impact of these 2 antioxidative defense genes on neurostructural changes, ideally integrating protein levels of antioxidative enzymes and neurocognitive testing. Such studies hold the potential of identifying individuals for whom antioxidant therapeutic approaches may be beneficial and identifying intermediate phenotypes for target engagement.

## Supplementary Material

pyab056_suppl_Supplementary_Tables
