# Supplementary material for: Antioxidative Defense Genes and Brain Structure in Youth Bipolar Disorder
Source: Int J Neuropsychopharmacol. 2021 Aug 13;25(2):89–98. doi: 10.1093/ijnp/pyab056 (PMC8832218; doi:10.1093/ijnp/pyab056)
Supplement: pyab056_suppl_Supplementary_Tables [file pyab056_suppl_supplementary_tables.docx]

**Supplementary Tables (S):**

**S1.** SOD2 ROIs Analyses Estimated Marginal Mean.

|  | Genotype Group | | | Diagnosis by Genotype Group | | | | | |
| --- | --- | --- | --- | --- | --- | --- | --- | --- | --- |
|  | AA | AG | GG | BD_AA_ | HC_AA_ | BD_AG_ | HC_AG_ | BD_GG_ | HC_GG_ |
| ROIs | (n=42) | (n=75) | (n=28) | (n=20) | (n=22) | (n=40) | (n=35) | (n=14) | (n=14) |
| cACC Area | 0.016 | 0.017 | 0.021 | 0.017 | 0.016 | 0.016 | 0.018 | 0.015 | 0.026 |
| cACC Volume | 0.047 | 0.050 | 0.073 | 0.051 | 0.043 | 0.048 | 0.053 | 0.045 | 0.101 |
| cACC Thickness | 2.656 | 2.701 | 2.874 | 2.642 | 2.669 | 2.679 | 2.725 | 2.713 | 3.035 |
| PFC Area | 0.516 | 0.524 | 0.559 | 0.490 | 0.541 | 0.534 | 0.514 | 0.512 | 0.060 |
| PFC Volume | 1.608 | 1.743 | 2.036 | 1.718 | 1.762 | 1.745 | 1.763 | 1.679 | 1.828 |
| PFC Thickness | 2.836 | 2.828 | 3.010 | 2.897 | 2.782 | 2.809 | 2.849 | 2.995 | 3.025 |
| Hippocampal Volume | 0.082 | 0.074 | 0.080 | 0.090 | 0.074 | 0.074 | 0.073 | 0.080 | 0.081 |
| cACC=caudal Anterior cingulate cortex; PFC= Prefrontal cortex; BD = Bipolar disorder; HC = Healthy control. Brain measurement units: area= mm^2^, volume= mm^3^, thickness=mm.  Note: the group mean for volume and area are scaled by 10^-5^. | | | | | | | | | |

**S2.** Mat-lab GPX3 ROIs Analyses Estimated Marginal Mean.

|  | Genotype Group | | Diagnosis by Genotype Group | | | |
| --- | --- | --- | --- | --- | --- | --- |
|  | AA/AC | CC | BD_AA/AC_ | HC_AA/AC_ | BD_CC_ | HC_CC_ |
| ROIs | (n=42) | (n=75) | (n=20) | (n=22) | (n=40) | (n=35) |
| cACC Area | 0.016 | 0.018 | 0.015 | 0.017 | 0.017 | 0.020 |
| cACC Volume | 0.047 | 0.056 | 0.044 | 0.048 | 0.050 | 0.062 |
| cACC Thickness | 2.661 | 2.773 | 2.625 | 2.690 | 2.723 | 2.837 |
| PFC Area | 0.487 | 0.549 | 0.428 | 0.533 | 0.549 | 0.548 |
| PFC Volume | 1.584 | 1.820 | 1.466 | 1.677 | 1.855 | 1.755 |
| PFC Thickness | 2.844 | 2.868 | 2.838 | 2.850 | 2.885 | 2.846 |
| Hippocampal Volume | 0.072 | 0.080 | 0.071 | 0.072 | 0.084 | 0.076 |
| cACC=caudal Anterior cingulate cortex; PFC= Prefrontal cortex; BD = Bipolar disorder; HC = Healthy control. Brain measurement units: area= mm^2^, volume= mm^3^, thickness=mm.  Note: the estimated marginal mean for volume and area are scaled by 10^-5^. | | | | | | |

**S3.** Results for Region of Interests Analyses.

|  |  |  | cACC  Area | | cACC  Volume | | cACC Thickness | | PFC  Area | | PFC  Volume | | PFC Thickness | | Hippocampal Volume | |
| --- | --- | --- | --- | --- | --- | --- | --- | --- | --- | --- | --- | --- | --- | --- | --- | --- |
| SNPs |  |  | F | *p* | F | *p* | F | *p* | F | *p* | F | *p* | F | *p* | F | *p* |
| *SOD2* rs4880 | Gene Main Effect |  | 1.89 | 0.16 | 5.28 | **0.01^*^** | 0.78 | 0.46 | 0.78 | 0.46 | 4.91 | **0.01**^*^ | 1.33 | 0.27 | 1.98 | 0.14 |
|  | Post-hoc | AA/AG | - | - | 0.36 | 0.55 | - | **-** | - | - | 1.62 | 0.21 | - | **-** | - | - |
|  |  | AG/GG | - | - | 8.19 | **0.005**^*^ | - | **-** | - | - | 5.51 | **0.02** | - | **-** | - | - |
|  |  | AA/GG | - | - | 9.69 | **0.002**^*^ | - | **-** | - | - | 9.79 | **0.002**^*^ | - | **-** | - | - |
|  | Interaction Effect |  | 3.37 | **0.04** | 7.50 | **0.001^*^** | 0.40 | 0.67 | 1.80 | 0.17 | 3.27 | **0.04** | 0.42 | 0.66 | 1.65 | 0.20 |
|  | Post-hoc | HC_AA_/HC_GG_ | 11.48 | **0.001**^*^ | 27.25 | **<0.001**^*^ | - | **-** | - | - | 2.68 | 0.10 | - | **-** | - | - |
|  |  | HC_AA_/HC_AG_ | 1.37 | 0.24 | 1.61 | 0.21 | - | **-** | - | - | 0.001 | 0.97 | - | **-** | - | - |
|  |  | HC_AG_/HC_GG_ | 7.24 | **0.008** | 20.49 | **<0.001**^*^ | - | **-** | - | - | 9.48 | **0.003**^*^ | - | **-** | - | - |
|  |  | BD_AA_/BD_GG_ | 0.22 | 0.64 | 0.19 | 0.66 | - | **-** | - | - | 0.93 | 0.34 | - | **-** | - | - |
|  |  | BD_AA_/BD_AG_ | 0.13 | 0.72 | 0.05 | 0.83 | - | **-** | - | - | 0.71 | 0.40 | - | **-** | - | - |
|  |  | BD_AG_/BD_GG_ | 0.04 | 0.84 | 0.07 | 0.77 | - | **-** | - | - | 0.12 | 0.73 | - | **-** | - | - |
|  |  | BD_AA_/HC_AA_ | 0.27 | 0.60 | 0.59 | 0.45 | - | **-** | - | - | 1.41 | 0.24 | - | **-** | - | - |
|  |  | BD_AG_/HC_AG_ | 0.92 | 0.34 | 0.31 | 0.58 | - | **-** | - | - | 0.40 | 0.24 | - | **-** | - | - |
|  |  | BD_GG_/HC_GG_ | 7.91 | **0.006**^*^ | 16.75 | **<0.001**^*^ | - | **-** | - | - | 11.46 | **0.001**^*^ | - | **-** | - | - |
| *GPX3* rs3792797 | Gene Main Effect |  | 2.29 | 0.13 | 2.67 | 0.11 | 0.07 | 0.79 | 7.29 | **0.008^*^** | 6.11 | **0.015*** | 1.10 | 0.30 | 4.01 | 0.048 |
|  | Interaction Effect |  | 0.12 | 0.71 | 0.46 | 0.50 | 0.04 | 0.83 | 4.44 | **0.04** | 2.18 | 0.14 | 0.11 | 0.75 | 1.10 | 0.30 |
|  | Post-hoc | HC_AA&AC_/HC_CC_ | - | - | - | - | - | - | 0.23 | 0.96 | - | - | - | - | - | - |
|  |  | BD_AA&AC_/BD_CC_ | - | - | - | - | - | - | 9.31 | **0.003^*^** | - | - | - | - | - | - |
|  |  | BD_AA&AC/_HC_AA&AC_ | - | - | - | - | - | - | 6.34 | **0.01^*^** | - | - | - | - | - | - |
|  |  | BD_CC_/HC_CC_ | - | - | - | - | - | - | 0.002 | 0.96 | - | - | - | - | - | - |
| cACC=caudal Anterior cingulate cortex; PFC= Prefrontal cortex; BD = Bipolar disorder; HC = Healthy control. Significant results are Bolded. *=Finding remains significant after correction for multiple comparisons. - = Not applicable due to the gene main effect or interaction effect not being significant. | | | | | | | | | | | | | | | | |

**S4:** SOD2 Whole Brain Gene Main Effect Post-hoc Analyses.

| Region | Estimated Marginal Mean | | | Post-hoc (F; *p*) | | |  |
| --- | --- | --- | --- | --- | --- | --- | --- |
|  | AA | AG | GG | AG/GG | AA/GG | AA/AG |  |
| lh superior frontal volume | 2.10 | 2.63 | 3.26 | 5.74; 0.02 | 17.12; <0.001 | 6.61; 0.01 |  |
| rh caudal middle frontal volume | 1.92 | 2.15 | 3.67 | 17.53; <0.001 | 20.48; <0.001 | 0.71; 0.40 |  |
| rh superior temporal volume | 1.87 | 2.06 | 3.34 | 14.53; <0.001 | 16.62; <0.001 | 0.50; 0.48 |  |
| rh superior temporal thickness | 2.98 | 3.02 | 3.77 | 23.47; <0.001 | 22.54; <0.001 | 0.14; 0.71 |  |
| Note: the estimated marginal mean for volume and area are scaled by 10^-5^. | | | | | | | |

**S5:** SOD2 Whole Brain Interaction Effect Post-hoc Analyses.

| Region | Estimated marginal mean | | | | | | Post-hoc (F; p) | | | | | | | | |
| --- | --- | --- | --- | --- | --- | --- | --- | --- | --- | --- | --- | --- | --- | --- | --- |
|  | BD_AA_ | BD_AG_ | BD_GG_ | HC_AA_ | HC_AG_ | HC_GG_ | HC_AA_/  HC_GG_ | HC_AA_/  HC_AG_ | HC _AG_/  HC _GG_ | BD_GG_/  HC_GG_ | BD_AG_/  HC_AG_ | BD_AA_/  HC_AA_ | BD_AA_/  BD_GG_ | BD_AA_/  BD_AG_ | BD_AG_/  BD_GG_ |
| rhparacentral area | 0.48 | 0.51 | 0.29 | 0.45 | 0.48 | 0.64 | 10.50;  0.002 | 0.48;  0.49 | 8.28;  0.005 | 25.27;  <0.001 | 0.42;  0.52 | 0.19;  0.66 | 8.59;  0.004 | 0.36;  0.55 | 14.00;  <0.001 |
| BD = Bipolar disorder; HC = Healthy control. Note: the estimated marginal mean for volume and area are scaled by 10^-5^. | | | | | | | | | | | | | | | |

**S6:** GPX3 Whole Brain Interaction Effect Post-hoc Analyses.

| Region | Estimated Marginal Mean | | | | Post-hoc (F; *p*) | | | |
| --- | --- | --- | --- | --- | --- | --- | --- | --- |
|  | BD_AA/AC_ | BD_CC_ | HC_AA/AC_ | HC_CC_ | BD_AA&AC_/BD_CC_ | BD_AA&AC_/HC_AA&AC_ | BD_CC_/HC_CC_ | HC_AA&AC_/HC_CC_ |
| lh superior frontal area | 0.415 | 0.634 | 0.623 | 0.521 | 8.12; 0.005 | 6.69; 0.01 | 4.20; 0.04 | 2.88; 0.09 |
| rh superior frontal area | 0.485 | 0.783 | 0.776 | 0.681 | 9.55; 0.003 | 8.26; 0.004 | 2.19; 0.14 | 1.58; 0.21 |
| rh supramarginal volume | 1.047 | 1.722 | 1.556 | 1.071 | 6.33; 0.01 | 3.27; 0.07 | 11.53; <0.001 | 5.35; 0.02 |
| BD = Bipolar disorder; HC = Healthy control. Note: the estimated marginal mean for volume and area are scaled by 10^-5^. | | | | | | | | |
